# Supplementary material for: Seminoma and Embryonal Carcinoma Footprints Identified by Analysis of Integrated Genome-Wide Epigenetic and Expression Profiles of Germ Cell Cancer Cell Lines
Source: PLoS One. 2014 Jun 2;9(6):e98330. doi: 10.1371/journal.pone.0098330 (PMC4041891; doi:10.1371/journal.pone.0098330)
Supplement: File S1 — ZIP file containing DMRforPairs output for significant regions. Please start from the html files. (ZIP) [file pone.0098330.s008.zip › figures/15592.pdf]

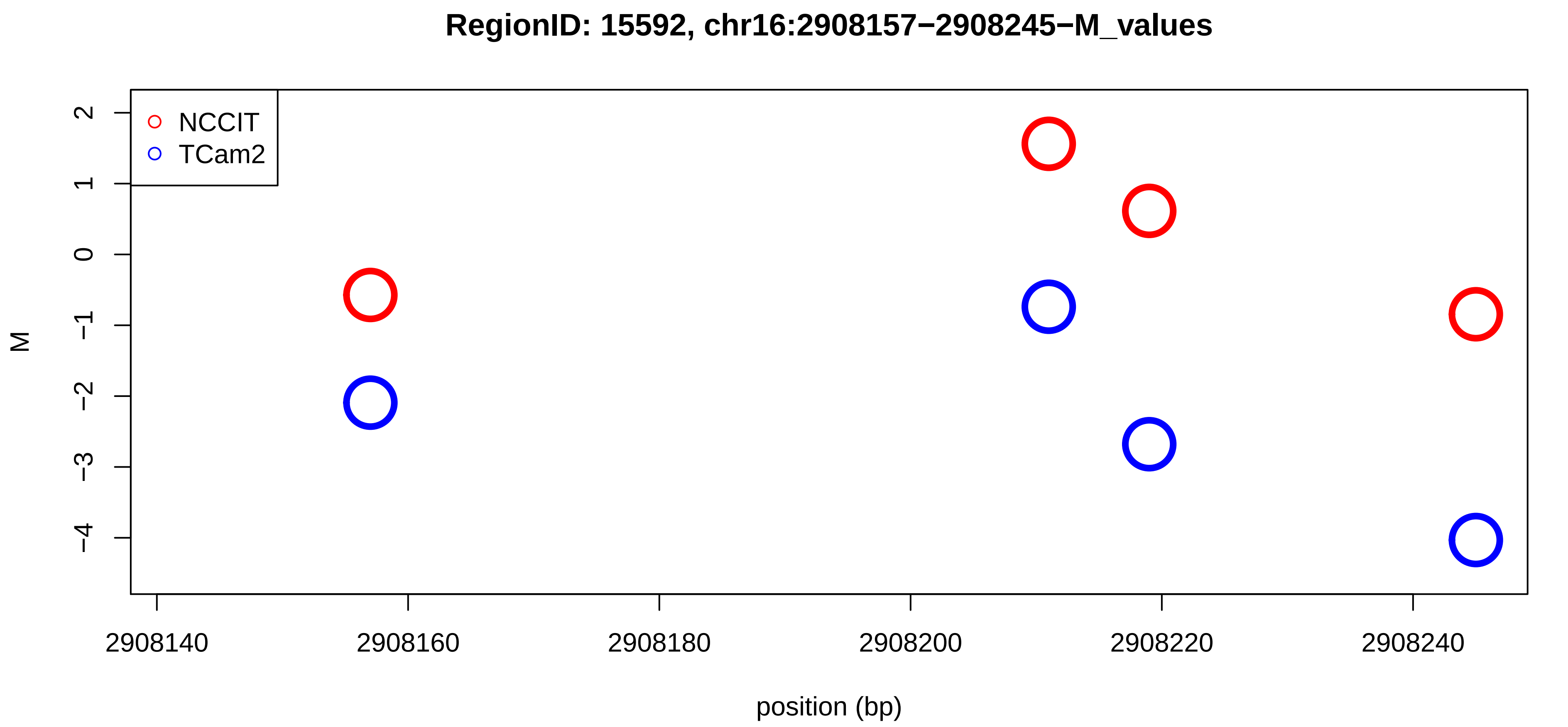

RegionID: 15592, chr16:2908157−2908245−M_values
NCCIT
TCam2
M
2
1
0
−1
−2
−3
−4
2908140
2908160
2908180
2908200
2908220
2908240
position (bp)

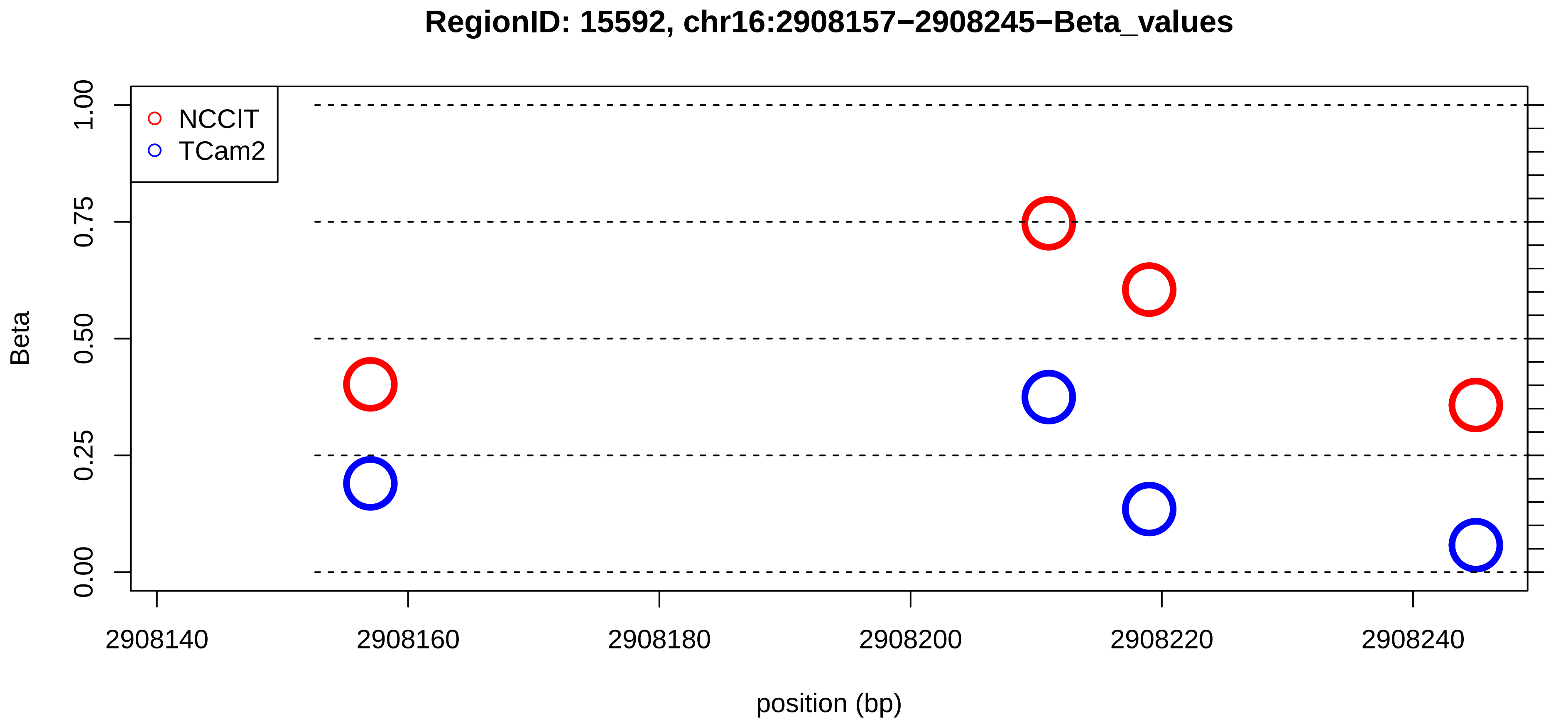

RegionID: 15592, chr16:2908157−2908245−Beta_values
NCCIT
TCam2
Beta
1.00
0.75
0.50
0.25
0.00
2908140
2908160
2908180
2908200
2908220
2908240
position (bp)
